# Co-registration of MALDI-MSI and histology demonstrates gangliosides co-localize with amyloid beta plaques in Alzheimer’s disease

**DOI:** 10.21203/rs.3.rs-3985371/v1

**Published:** 2024-02-27

**Authors:** Nikita Ollen-Bittle, Shervin Pejhan, Stephen H. Pasternak, C Dirk Keene, Qi Zhang, Shawn N Whitehead

**Affiliations:** 1 Department of Anatomy and Cell Biology, Schulich School of Medicine and Dentistry, Western University, London, Ontario, N6A 5C1; 2 Robarts Research Institute, Schulich School of Medicine and Dentistry, London, ON, Canada; 3 Department of Clinical Neurological Sciences, Schulich School of Medicine and Dentistry, Western University, London, Ontario, N6A 5C1.; 4 Department of Pathology and Laboratory Medicine, London Health Sciences Centre, London, ON, Canada; 5 Department of Laboratory Medicine & Pathology, Division of Neuropathology, University of Washington School of Medicine, Seattle, WA, USA

**Keywords:** Alzheimer’s disease, gangliosides, lipids, MALDI-MSI, digital pathology

## Abstract

Alzheimer’s disease (AD) is a progressive neurological condition characterized by impaired cognitive function and behavioural alterations. While AD research historically centered around mis-folded proteins, advances in mass spectrometry techniques have triggered increased exploration of the AD lipidome with lipid dysregulation emerging as a critical player in AD pathogenesis. Gangliosides are a class of glycosphingolipids enriched within the central nervous system. Previous work has suggested a shift in a-series gangliosides from complex (GM1) to simple (GM2 and GM3) species may be related to the development of neurodegenerative disease. Additionally, complex gangliosides with 20 carbon sphingosine chains have been shown to increase in the aging brain. In this study, we utilized matrix assisted laser desorption ionization mass spectrometry imaging (MALDI-MSI) to interrogate the *in situ* relationship of a-series gangliosides with either 18 or 20 carbon sphingosine chains (d18:1 or d20:1 respectively) in the post-mortem human AD brain. Here, we expanded upon previous literature and demonstrated a significant decrease in the GM1 d20:1:GM1 d18:1 ratio in regions of the dentate gyrus and entorhinal cortex in AD relative to control brain tissue. Then we demonstrated that the MALDI-MSI profile of GM3 co-localizes with histologically confirmed amyloid beta (Aβ) plaques and found a significant increase in both GM1 and GM3 in proximity to Aβ plaques. Collectively these results support past literature and demonstrate a perturbation of the ganglioside profile in AD. Moreover, this work validates a pipeline for MALDI-MSI and classic histological staining in the same tissue sections. This demonstrates feasibility for integrating untargeted mass spectrometry imaging approaches into a digital pathology framework.

## Introduction

Alzheimer’s disease (AD) is a devastating neurodegenerative condition clinically characterized by progressive memory loss, declining cognitive function and behavioural alterations. AD is the most common cause of dementia with an estimated 9.9 million new cases annually throughout the world[30]. Neuropathologically, AD is characterized by amyloid beta (Aβ) plaque deposition and aggregates of hyperphosphorylated tau protein (neurofibrillary tangles, NFT) with associated synaptic degeneration and ultimately cell death. For most individuals, the degenerative process of AD follows a predictable pattern, which is the basis for the neuropathological staging of the disease, which incorporates Aβ plaque distribution across the brain (Thal Phase), NFT distribution across the brain (Braak stage[8]), and cerebral cortical neuritic plaque density (CERAD score)[18]. One theory suggests AD tau-pathology originates within the transentorhinal and entorhinal cortex (EC) before spreading to subsequent regions. Alternatively, another proposes degeneration first starts in nonthalamic subcortical nuclei with cortical projections such as the locus coeruleus and the nucleus basalis of Meynert, a basal forebrain (BF) structure[3]. Braak stages I/II identify NFTs predominantly in the EC and proximal areas, stages III/IV identify NFTs more predominantly within the hippocampus, amygdala and slightly into association cortex and finally stages V/VI identify NFTs throughout much of the neocortex[8]. Meanwhile, studies assessing genetic data, as well as autopsy and neuroimaging results have indicated that Aβ plaque pathology precedes cortical tau pathology and may in part drive tau pathology[19]. Currently, both the staging process and our understanding of AD neuropathology is largely protein-centric.

The reason mis-folded proteins have been the primary focus of AD literature is two-fold: 1) abnormal mis-folded protein accumulation driving the Aβ hypothesis has dominated the field of AD research, and 2) lipid research has been limited by a lack of analytical imaging techniques capable of detecting complex lipid species *in situ*. However, lipid dysregulation is now being recognized as a potential driver of AD progression. Lipid peroxidation occurs early in AD pathogenesis[9], and lipid droplets, indicative of altered lipid processing, accumulate in the AD brain[16]. Moreover, polymorphisms of the apolipoprotein E (*APOE*) gene, by far the strongest genetic risk factor for sporadic (non-autosomal dominant AD), alters ApoE’s ability to mediate lipid transport, metabolism, and overall lipid homeostasis[36]. More work is needed to elucidate the complex interplay of both the proteome and lipidome in AD pathogenesis.

Gangliosides represent a lipid class of glycosphingolipids that are particularly enriched within the central nervous system and have been implicated in a variety of neurodegenerative conditions including AD[7, 17]. Gangliosides have been demonstrated to function as lipid rafts within cellular membranes and mediate membrane organization, neurotransmission, and neurogenesis[32]. Moreover, soluble Aβ has a high affinity for ganglioside-containing lipid rafts[33], and gangliosides are thought to play a critical role in altering the secondary structure of Aβ[24]. It has been proposed that a shift from complex neuroprotective GM1 species to simple GM2 and GM3 species, is linked to neurodegeneration^4^. However, the relationship of ganglioside species and AD pathogenesis is complex. While GM1 has been shown to exert neuroprotective effects[15], it has also been shown to generate a toxic secondary structure of Aβ[27]. Given the conflicting reports of the role of gangliosides in AD pathogenesis and that GM1 has been administered to human patients with reported benefits including patients becoming more active[5], it is critical we increase our understanding of the spatial localization of both simple and complex gangliosides in the AD brain, so we can better understand how the lipidome interacts with established protein hallmarks of AD progression.

Moreover, the ratio of GM1 d20:1 to GM1 d18:1 has been shown to increase with age in both animals[12] and humans[34] and be perturbed in post-mortem human AD brain tissue[17]. Therefore, although they only vary by two carbons, it is critical that we explore the spatial localization of these ganglioside species in AD relative to control tissues, as a shift towards increased GM1 d20:1 in distinct memory circuits in the brain may reflect a molecular maturation requisite for healthy aging. To overcome this gap in knowledge we must utilize technology able to detect lipid-based molecular profiles without tissue homogenization.

**Figure 1.**
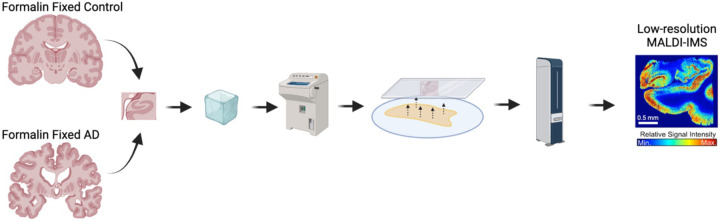
Workflow of tissue preparation for formalin-fixed tissue. Regions of interest were first blocked from formalin-fixed AD and control brains. Tissue blocks were then flash frozen over dry ice and prepped for MALDI-MSI. Blocks were sectioned on a cryostat to 10 μm, mounted on an ITO coated slide and washed with ammonium formate prior to DAN matrix sublimation. MALDI-MSI was then run with a laser step distance of 70 μm. Created with BioRender.com.

**Figure 2.**
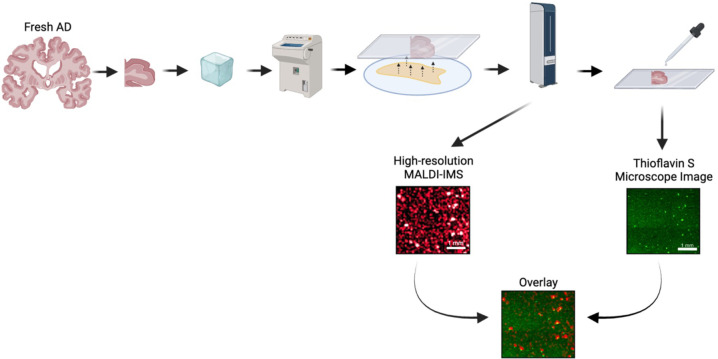
Pipeline for overlay of MALDI-MSI with traditional histology. The tissue preparation for fresh frozen samples followed the same protocol as formalin-fixed tissue up to the point of MALDI-MSI. In brief, tissue was first blocked then flash frozen. The tissue was then sectioned to 10 μm, mounted on ITO coated slides and washed with ammonium formate prior to DAN matrix sublimation. High resolution MALDI-MSI scans with a laser step distance of 40 μm were then run on prepared sections. Following the MALDI-MSI scan the same slide was stained with thioflavin S for amyloid beta plaques. A microscope image of the thioflavin S stain was overlayed with the high resolution MALDI-MSI scan by aligning tissue borders and distinct tissue imperfections (tears) purposely imaged to allow for accurate overlay. Created with BioRender.com.

Matrix assisted laser desorption ionization mass spectrometry imaging (MALDI-MSI) is a technique that allows for the untargeted spatial visualization of biomolecules across a tissue sample. MALDI-MSI is uniquely equipped to detect and measure lipids *in situ*, as it is capable of detecting slight changes in molecular structure (i.e. d18:1 vs d20:1 ganglioside species) and is not impeded by their small molecular size or dynamic structural nature[37]. Herein, we utilized MALDI-MSI to assess changes in gangliosides within the post-mortem human AD brain. Based on previous literature[10, 11], we hypothesized the AD brain would demonstrate a widespread increase in simple ganglioside species throughout anatomical regions associated with AD pathogenesis; however, we revealed a much more complex relationship. First, we evoked a lower resolution technique to survey both simple and complex ganglioside species in formalin-fixed AD compared to control brain tissue, in regions including: the dentate gyrus (DG) and cornu amonis (CA) of the hippocampus, the EC, the BF, the forebrain white matter underlying the EC and the anterior commissure (AC) (Figure 1). We then utilized a high-resolution scan overlayed with histological staining of Aβ to demonstrate plaque vs. plaque-adjacent ganglioside profiles in fresh frozen brain (Figure 2). Overall, we show ganglioside enrichment occurs at the site of Aβ plaques, and the metabolism of d20:1 ganglioside species may be particularly perturbed in AD resulting in a shift towards GM3 d20:1.

## Materials and Methods

### Tissue Samples and Research Ethics

#### Formalin-fixed samples:

AD brain and control samples were obtained through the Pathology Department at the London Health Sciences Centre, Ontario, Canada, approved by Western University Research Ethics Board (protocol 108492). The brain tissue was stored in 10% formalin until processed for experimentation. Donors were selected based on neuropathological diagnoses and included five AD and five control brains. The AD cohort included blocks from one male and four female patients with a mean age of 78.6 years. The control cohort for hippocampal and EC samples included blocks from three male and two female donors, with a mean age of 71 years, whereas the control cohort for BF and AC samples included blocks from three male and two female donors with a mean age of 69.8 years.

#### Fresh frozen samples:

Tissues for the plaque/plaque adjacent analysis were obtained through the BioRepository and Integrated Neuropathology (BRaIN) laboratory and the Precision Neuropathology Core at the University of Washington (UW) in Seattle, Washington, USA. The BRaIN Lab supports diverse prospective cohort studies of brain aging and dementia, with tissues used in this study derived from participants in the UW Alzheimer’s Disease Research Center (ADRC), the Kaiser Permanente Washington Health Research Institute Adult Changes in Thought (ACT) Study, and the Seattle Longitudinal Study (SLS). The BRaIN Lab has developed a rapid autopsy protocol that includes methods for rapid, thin slicing of fresh brain and slab freezing, which was performed on the samples used in this study. Donors were selected for neuropathologically-confirmed AD dementia and included one male and three female donors with mean age at death of 88.3 years (range 70–98 yrs) and average post-mortem interval to tissue freezing of 7.2 hours (range 5.0–9.8 hrs). Donors were neuropathologically confirmed to be negative for Lewy body disease, vascular brain injury, frontotemporal lobar degeneration, and hippocampal sclerosis. One donor was positive for stage 1 and stage 2 limbic-predominant, age-related TDP-43 encephalopathy (LATE), which is commonly associated with ADNC.

### Tissue Blocking

All tissue blocking of formalin-fixed samples was performed by the Pathology department at the London Health Sciences Centre, Ontario, Canada. Regions of interest including the: hippocampus, entorhinal cortex, basal forebrain and anterior commissure were blocked from post-mortem formalin-fixed AD and control brains to fit into standard pathology sized cassettes. Dissection of fresh frozen dorsolateral prefrontal cortex samples were performed by UW BRaIN Lab to fit into standard pathology sized cassettes.

### Tissue Sectioning

The tissue blocks were flash frozen over dry ice. Following flash-freezing tissue blocks were stored at −80 °C until sectioning. Tissue was then secured to a cryostat chuck by freezing the base of the tissue to the chuck using deionized H_2_O (dH_2_O). Once secured to the chuck the tissue was allowed to acclimate in the cryostat at −21 °C for 15 minutes prior to sectioning. The tissue was then sectioned to 10 μm using a cryostat (Thermo-Fisher Scientific CryoStar NX50, Toronto, Canada). The sections were then thaw-mounted onto indium tin oxide (ITO) coated slides. Formalin-fixed AD and control samples were paired by most comparable Brodmann landmarks and prepared and run on the same slides.

### Ammonium Formate Wash

Tissue sections were washed in 50 mM ammonium formate for 30 seconds to enhance ganglioside signal as previously described[2]. The wash was applied over slides sitting tissue side up on a flat surface to prevent tissue displacement and the slide was gently tilted to allow runoff at 30 seconds. Slides were then placed in a desiccator for 10 minutes/until all moisture was visibly evaporated off the slide, prior to matrix application.

### Matrix Application

1,5-diaminonapthalene (DAN) matrix was applied to slides as previously described[4]. In brief, 300 mg of DAN matrix was placed on the bottom surface of the sublimation apparatus and the side was secured above. The apparatus was secured over a hotplate stabilized to 140 °C. Under vacuum pressure the matrix was allowed to sublimate over the tissue for seven minutes ensuring an even and sufficiently thick layer of matrix deposited over the tissue.

### MALDI-MSI

A Sciex 5800 TOF/TOF system, MALDI TOF/TOF (Sciex, Framingham, MA, USA) with a 349 nm Nd:YLF OptiBeam On-Axis laser with a pulse rate of 400 Hz was utilized for all MALDI-MSI data collected. All scans were conducted in reflectron negative mode and were acquired with a mass range of m/z 1000−2000. For low resolution scans a laser step distance of 70 μm was utilized. For high resolution scans a laser step distance of 40 μm was utilized.

### Histology

#### H&E:

All H&E staining was performed on subsequent sections to those used for MALDI-MSI and was done on positively charged slides. Hematoxylin was applied to slides for 1.5 minutes. This was followed by two, 20 second washes in fresh tap water. Differentiation solution was then applied for 60 seconds, followed again by two, 20 seconds washes in fresh tap water. Scott’s Tap Water Substitute was then applied for 60 seconds followed by two, 20 second washes in fresh tap water. Eosin was then applied for 10 seconds. Dehydration was then preformed with standard serial washing in ethanol and finally the tissue was allowed to sit in xylene for 15 minutes prior to coverslipping.

#### Thioflavin S:

Thioflavin S staining was done directly on tissue sections used for MALDI-MSI on ITO coated slides after MALDI-MSI. Slides were first dipped in methanol and to remove DAN matrix and allowed to dry. Tissue was then fixed in 3% formaldehyde in PBS for 15 minutes. 1% Thioflavin S was made in 50 mL H_2_O and filtered prior to application. Tissue was first washed three times in 50% ethanol for three minutes each time. A three min H_2_O wash was then performed. 1% filtered thioflavin S was then applied to the samples for 10 minutes in the dark. All steps here on out were performed in the dark to preserve fluorescence. The tissue was then dehydrated using three, five-minute washes in 70% ethanol and three, three-minute washes in 50% ethanol. The tissue was then washed in dH_2_O for 15 minutes then allowed to air-dry for 15 minutes prior to coverslipping with Fluoroshield with DAPI.

### Microscope Images

Microscope images were taken using an upright brightfield microscope (Nikon Eclipse Ni-E, Nikon DS Fi2 colour camera, NIS Elements Imaging; Mississauga, ON). Stitched images were captured using a 10x objective lens.

### Region of Interest Selection

Anatomical region of interest (ROI) selection for comparisons between post-mortem AD and control brains were selected through use of H&E staining of hippocampus tissue sections taken adjacent to those used for MALDI-MSI and through use of the Adult Human Allen Brain Atlas[13]. ROI selection for plaque/plaque adjacent regions in AD brains were done through overlaying the thioflavin S staining with the MALDI-MSI scan on CorelDraw Software. Histology images and MALDI-MSI scans were overlayed by aligning tissue borders and distinct imperfections/tears in the tissue. MALDI-MSI scans were then set to transparent red or blue so only high intensity regions were visualized over the thioflavin S images.

### Data Analysis and Statistics

Following ROI selection, ganglioside peaks of interest in the mass spectra generated by MALDI-MSI were analyzed. Following baseline correction, the area under the curve of the three largest isotopic peaks for each species of interest was calculated as a ratio to the total area of the spectrum, as has been previously published[11, 12, 28, 35]. All data was checked for normality with a Shapiro-Wilk test. Non-paired data (GM1 and GM2 ratios between post-mortem AD and control brains) were analyzed with either a student’s t-test with Welch’s correction or a Mann-Whitney test, for parametric and nonparametric data respectively. Paired data (non-ratio comparisons between AD and control brains as well as plaque and plaque adjacent regions within AD brains) were analyzed with either a paired t-test or a Wilcoxon matched-pairs signed rank test for parametric and non-parametric data respectively. For plaque and plaque adjacent analysis three plaque regions and three adjacent regions were selected from each tissue block.

### Software

MALDI-MSI images were visualized, and ROIs were selected using Tissue-View software (Sciex). Graphs were generated using GraphPadPrism10 software (GraphPad Software Inc.). Results figures were created using CorelDRAW software. Workflow figures were created with BioRender.com.

## Results

**Figure 3.**
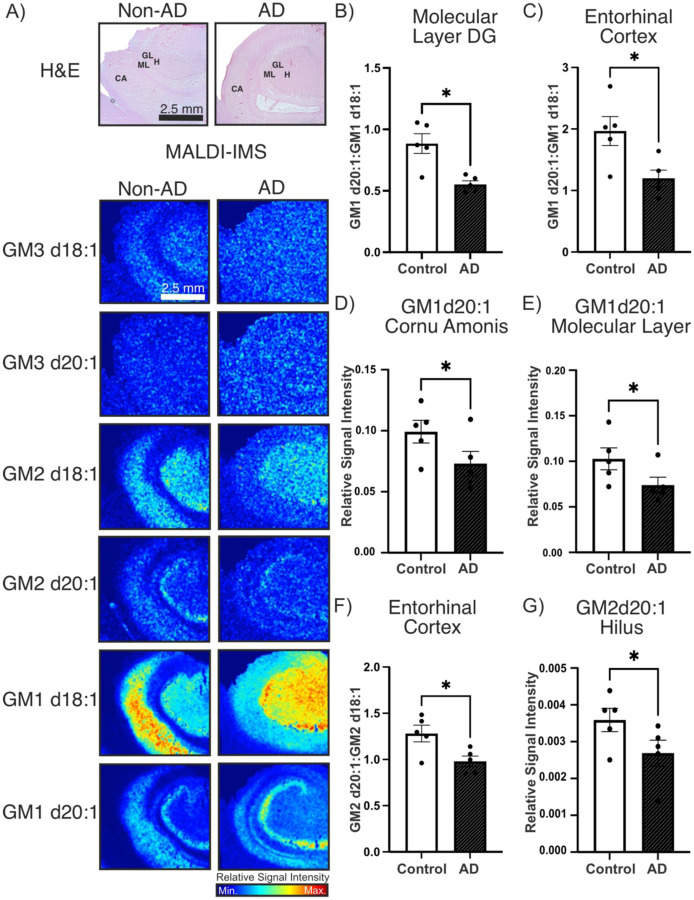
MALDI-MSI of formalin-fixed AD and control brains. A) MALDI-MSI “heat maps” visualized differential spatial localization of gangliosides in the hippocampus of AD compared to control brains. B) A significant decrease in the GM1 d20:1:GM1 d18:1 ratio was observed in the ML of the DG (n=5, p = 0.0114); C) as well as in the EC (n=5, p = 0.0279). D) A significant decrease in GM1 d20:1 was found in the CA (n=5, p = 0.0487) of the hippocampus; E) and the ML of the DG (n=5, p = 0.0305) in AD tissue compared to control tissue. F) A significant decrease (n=5, p = 0.0264) in the GM2 d20:1:GM2 d18:1 ratio was identified in the EC of AD samples. G) A significant decrease (n=5, p = 0.0194) was found in GM2 d20:1 in the H of AD compared to control tissue. CA: cornu amonis, ML: molecular layer, GL: granular layer, H: hilus.

First, low-resolution MALDI-MSI scans of the formalin-fixed brain tissue from neuropathologically diagnosed AD brains (n=5) and control (n=5) brains were assessed. Regions of interest included the hilus (H), granular layer (GL) and molecular layer (ML) of the DG, the CA of the hippocampus, the EC, forebrain white matter subcortical to the EC, the BF and the AC. Low resolution scans allowed for the mass spectrometry imaging of an entire cryostat cut section to visualize the ganglioside profile across the entirety of the tissue block. Visually, gangliosides appeared to have a differential profile in the hippocampus of AD brains compared to control brains as demonstrated in Figure 3a. Examining the GM1 d20:1:GM1 d18:1 ratio in the regions of interest, we identified a significant decrease (p = 0.0114) in the GM1 d20:1:GM1 d18:1 ratio in the ML of the DG as well as in the EC (p = 0.0279) (Figure 3b,c). We further identified a significant decrease in the GM1 d20:1 species in the ML of the DG (p = 0.0305) and in the CA (p = 0.0487) (Figure 3d,e). Moving on to the simple ganglioside species we assessed the GM2 d20:1:GM2 d18:1 ratio and found a significant decrease (p = 0.0264) in this ratio in the EC (Figure 3f). Additionally, we found a significant decrease (p = 0.0194) in GM2 d20:1 in the H of the DG (Figure 3g). Additional results from brain regions where no significant results were obtained can be found in **Supplemental Figures 1–3**. GM3 species throughout brain regions of interest and GM2 species in the AC were not analyzed at this stage because the signal to noise ratio of these peaks was too low for accurate comparisons. However, this led us to note that simple gangliosides were appearing in a punctate pattern more prominently in the grey matter of AD brain tissue.

**Figure 4.**
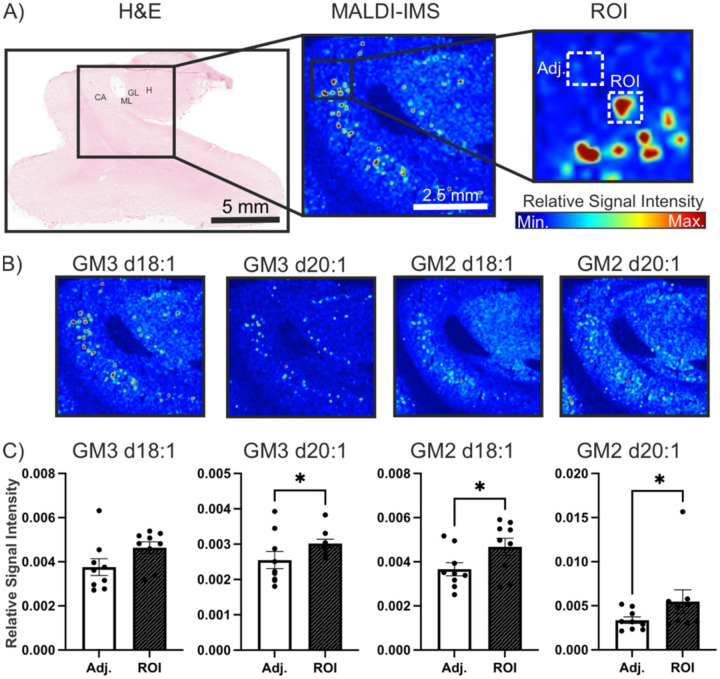
Simple gangliosides localize in a punctate pattern in low resolution MALDI-MSI of AD samples. A) MALDI-MSI ROI’s were selected around punctate hotspots of simple gangliosides. B) GM2 and GM3 species appeared in this pattern in AD samples. C) A significant increase in GM3 d20:1 (n=5, p = 0.0136), GM2 d18:1 (n=5, p = 0.0078), and GM2 d20:1 (n=5, p = 0.0234) was found in these punctate regions relative to adjacent regions. CA: cornu amonis, ML: molecular layer, GL: granular layer, H: hilus.

We noted that this punctate pattern of simple gangliosides was most pronounced in the grey matter of three AD donor tissue samples compared to the controls. From these three samples, we selected regions of interest around three of the punctate spots (n=9 regions of interest) and around three adjacent regions (n=9 adjacent regions) (Figure 4a,b). Analysis revealed a significant increase in GM3 d20:1 (p = 0.0136), GM2 d20:1 (p = 0.0234) and GM2 d18:1 (p = 0.0078) in the punctate regions of interest relative to the adjacent regions (Figure 4c). There was no significant increase in GM3 d18:1 (p = 0.0742), GM1 d18:1 (p = 0.1282) or GM1 d20:1 (0.0686).

Given the punctate pattern of simple gangliosides mirrored the known punctate appearance of Aβ plaques in AD, we wanted to investigate how these ganglioside profiles aligned with Aβ plaques. To obtain the best MALDI-MSI signal, while retaining tissue integrity for staining, we developed a workflow to post-fix and stain fresh frozen AD tissue after MALDI-MSI. Fresh frozen samples are the gold standard for MALDI-MSI as formalin fixation can cause protein crosslinking and subsequent ion suppression of desired molecules of interest. The fresh frozen AD samples also boasted better fluorescence for thioflavin S staining post MALDI-MSI. Of note, thioflavin S staining of the formalin-fixed tissue was attempted; however, the tissue exhibited poor fluorescence and generally poor tissue integrity causing the tissue to float off the slide in the staining process. We then attempted immunohistochemistry (IHC) of formalin-fixed tissue sections that were sectioned sequentially to the tissue section used for MALDI-MSI. While this was achieved (n = 1, **Supplemental Figure 4**), the tissue integrity of frozen sections made this a difficult process and since the IHC was done on a tissue section adjacent to the MALDI-MSI section instead of the same section, the novel workflow of post-fixation of fresh frozen samples and staining tissue after MALDI-MSI was utilized.

**Figure 5.**
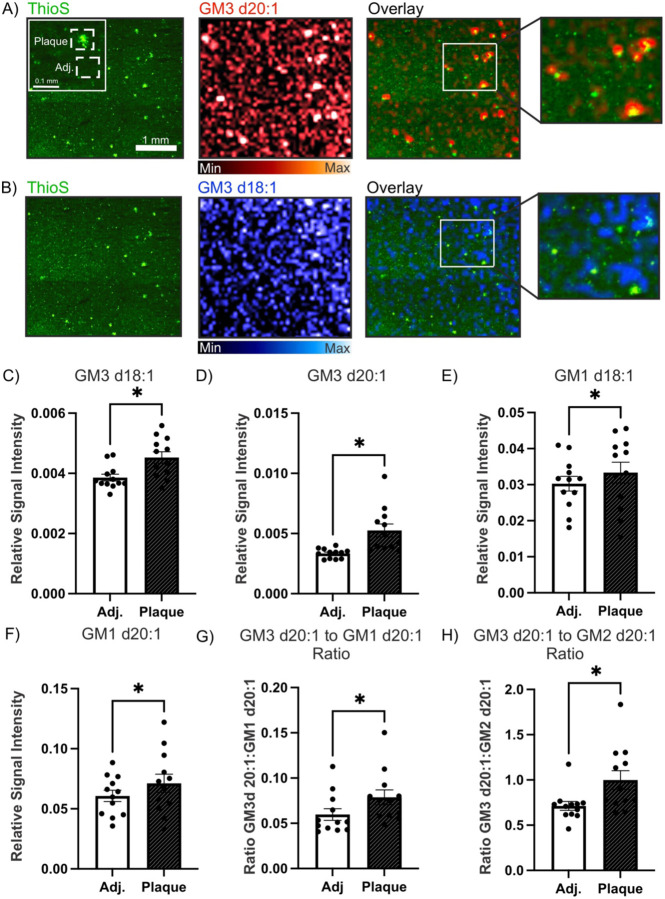
GM3 localizes with amyloid beta plaques in the AD brain. High resolution MALDI-MSI of A) GM3 d20:1 and B) GM3 d18:1 overlayed with thioflavin S staining of amyloid beta plaques in fresh frozen AD brain samples. C) GM3 d18:1 (n=12, p = 0.0033), D) GM3 d20:1 (n=12, p = 0.0005), E) GM1 d18:1 (n = 12, p = 0.0360), and F) GM1 d20:1 (n=12, p = 0.0367) were all found to significantly increase in plaque relative to plaque adjacent regions in the AD brain. Within the plaque regions there was an increase in G) GM3 d20:1:GM1 d20:1 (n=12, p = 0.0010), and H) GM3 d20:1:GM2 d20:1 ratio (n=12, p = 0.0015), compared to plaque adjacent regions.

High resolution MALDI-MSI was used to examine distinct regions of fresh frozen AD brains (n = 4) with a high neuropathologically confirmed plaque load. Following the high resolution MALDI-MSI scan the same tissue section that had undergone MALDI-MSI was then stained with thioflavin S for Aβ plaques. Three regions of interest were selected around the plaques and three regions of interest adjacent to the plaques were selected from each of the scans for paired analysis (plaques n = 12, adjacent n=12). Visually, GM3 d20:1 (Figure 5a) and GM3d 18:1 (Figure 5b) appeared to co-register with the Aβ plaques. A significant increase was found in GM3 d20:1 (p = 0.0005), GM3 d18:1 (p = 0.0033), GM1 d20:1 (p = 0.0367) and GM1 d18:1 (p = 0.0360) in the plaque regions of interest relative to the plaque adjacent regions (Figure 5c–f). We then explored ratios between the GM3 species and the GM2 and GM1 species in the plaque regions compared to the plaque adjacent regions. While there was no significant difference in the 18 carbon species, there was a significant increase in the GM3 d20:1:GM1 d20:1 (p = 0.0010) and the GM3 d20:1:GM2 d20:1 (p = 0.0015) ratios in the plaque relative to the plaque adjacent regions (Figure 5g,h).

## Discussion

We report three critical outcomes from this study. Frist, we validate a workflow that successfully integrates both histological staining and MALDI-MSI on the same tissue section in human brain samples which will greatly enhance the integration of mass spectrometry imaging into digital pathology. Second we add to the current literature[12, 17, 35] on GM1 d20:1:GM1 d18:1 ratios in AD by elucidating a significant decrease in the EC, a brain region that was previously unexplored. Finally, we elucidate an intriguing ganglioside profile at Aβ deposits in the human brain, shedding light on the AD lipidome and providing a new lipid-based mechanism to interrogate the pathophysiologic progression of AD.

Previous studies have reported an age-related increase in GM1 d20:1 species with age in both rodent and human brain[12, 31, 35]. Previously, this relationship has been shown to be disrupted in AD[34] and specifically in the outer layer of the ML of the DG in AD[17]. Our results validate these findings by demonstrating a decrease in GM1 d20:1:GM1 d18:1 ratio in the ML and adds to this literature by further elucidating a decrease in the EC. We also demonstrate a decrease in the GM2 d20:1:GM2 d18:1 ratio in the EC as well as a decrease in d20:1 subspecies of both GM1 and GM2 in different regions of the hippocampus. These findings support a disturbance in d20:1 ganglioside species in the perforant pathway, of which projections from the EC terminate in the ML of the DG. Disturbances to this pathway have been critically implicated in AD[25] and this work provides greater insight into the molecular changes in AD pathology.

Utilizing high resolution MALDI-MSI also allowed us to interrogate the relationship of ganglioside disturbances with Aβ deposits. These results suggest GM3 and GM1 accumulation colocalizes with Aβ plaques in the brain and there may be a shift driving metabolism specifically towards GM3 d20:1 species in these regions. Previous work has demonstrated an increase in simple gangliosides (GM2 and GM3 species) in both mouse models and human AD[6, 10, 22]. Moreover, a multi-modal MALDI-MSI approach has previously been used to interrogate the Aβ plaque associated lipid profile in a transgenic mouse model of AD[21]. This work similarly demonstrated a plaque associated accumulation of GM3 d18:1 and GM3 d20:1, and differentially to our results also showed an increase in GM2 d18:1 and no significant increase in GM1 species[21]. In both results it is clear GM3 species co-localize with Aβ plaques. The differences may be accounted for by their higher 10 μm resolution, however differences in plaque heterogeneity and stage of disease should also be considered. Human plaques may also demonstrate differential structural and physiochemical variability compared to animal models[23]. Additionally, if we follow the hypothesis that an overall shift towards GM3 is occurring, this may be more predominant in end-stage disease as seen in the human tissue. More recently, MALDI-MSI and fluorescent staining of Aβ plaques was accomplished on post-mortem human tissue samples from AD patients with known presenilin 1 mutations[26]. These results similarly showed a correlation between MALDI-MSI signal intensity of gangliosides and histology confirmed Aβ plaque deposits, but also revealed vast heterogeneity in plaque associated lipids between patients. Previous work has hypothesized that an increase in simple gangliosides in AD may be due to accelerated lysosomal degradations and astrogliosis[22]. Further studies are needed to understand the cellular mechanisms driving these ganglioside changes associated with Aβ plaques. Collectively, these results support previous findings of a disturbance in the ganglioside profile in AD and highlight intriguing biologic changes occurring around Aβ plaques.

This study has certain limitations. Foremost, formalin-fixed AD samples were neuropathologically diagnosed and we do not have access to the clinical data reflecting the cognitive function of the donors from which the samples were obtained (although the fresh frozen sample cohort were all clinically and neuropathologically confirmed to have AD dementia using current consensus guidelines). It is well established that accumulation of Aβ plaques can occur in the brain of otherwise healthy or cognitively normal patients years and likely decades before the onset of dementia[1]. Additionally, this study was not powered to adequately assess the effects of age, sex or other comorbidities. Therefore, we emphasize that this work serves as a first step to increase our understanding of lipidomic changes associated with common neuropathologic changes found in AD, and while ganglioside disturbances may hold significant clinical relevance, more work is needed to understand this relationship. Finally, we utilized 40 μm resolution for high resolution MALDI-MSI scans. Previous literature examining the lipid profile associated with Aβ plaques in the mouse brain using both 10 and 30 m resolution, reported that Aβ plaque localization for some lipid species can only be conclusively observed at 10 μm resolution[21]. Therefore, we cannot rule out that higher resolution may have resulted in other ganglioside species exhibiting a significant association with Aβ plaques.

Perhaps most significantly, this paper develops, validates and, successfully demonstrates a workflow allowing both histological staining and MALDI-MSI to be conducted on the same tissue section in post-mortem human brain samples. Unlike antibody-based approaches to *in situ* visualization of molecules in post-mortem brain sections, MALDI-MSI offers an untargeted approach to molecular mapping that is specifically optimized for the identification of lipids. Previous work has utilized multi-modal MALDI-MSI to visualize both the protein plaques and lipids[21] and has even demonstrated histological co-registration of Aβ plaques in rodent samples[20]; however human tissue often exhibits integrity issues not seen in rodent samples since it is frequently preserved and stored for a significantly longer period of time prior to data collection. Therefore this work serves to validate recent advances in the use of MALDI-MSI and histological staining on the same tissue section in post-mortem human brain tissue[26] while further exhibiting how this technique can be used to interrogate specific biological questions through analysis of ratios between related lipid species. In contemporary medicine we are moving towards digital pathology. Our lab has previously established a macro to micro-scale imaging pipeline to overlay MRI and histology with MALDI-MSI[14, 29]. Integrating histology of the same tissue section used for MALDI-MSI into this pipeline will further enhance our understanding of how molecular changes drive tissue level pathology and may prove critical to biomarker and therapeutic target discovery. This manuscript demonstrates the feasibility of integrating untargeted imaging mass spectrometry approaches with classic histological staining in post-mortem human tissue. This has the potential to elucidate massive *in situ* datasets of molecules that correlate with known histological hallmarks of disease. This in turn would generate novel mechanisms of disease progression and could aid in pathology diagnosis.

## Data Availability

Data available upon request.
